# CDK4/6 inhibitors: a novel strategy for tumor radiosensitization

**DOI:** 10.1186/s13046-020-01693-w

**Published:** 2020-09-15

**Authors:** Yilan Yang, Jurui Luo, Xingxing Chen, Zhaozhi Yang, Xin Mei, Jinli Ma, Zhen Zhang, Xiaomao Guo, Xiaoli Yu

**Affiliations:** 1 Department of Radiation Oncology, Fudan University Shanghai Cancer Center, 270 DongAn Road, Shanghai, 200032 China; 2 Department of Oncology, Shanghai Medical College, Fudan University, Shanghai, 200032 China

**Keywords:** CDK4/6 inhibitor, Radiotherapy, Radiosensitivity

## Abstract

Recently, the focus of enhancing tumor radiosensitivity has shifted from chemotherapeutics to targeted therapies. Cyclin-dependent kinase 4 and 6 (CDK4/6) inhibitors are a novel class of selective cell cycle therapeutics that target the cyclin D-CDK4/6 complex and induce G1 phase arrest. These agents have demonstrated favorable effects when used as monotherapy or combined with endocrine therapy and targeted inhibitors, stimulating further explorations of other combination strategies. Multiple preclinical studies have indicated that CDK4/6 inhibitors exhibit a synergistic effect with radiotherapy both in vitro and in vivo. The principal mechanisms of radiosensitization effects include inhibition of DNA damage repair, enhancement of apoptosis, and blockade of cell cycle progression, which provide the rationale for clinical use. CDK4/6 inhibitors also induce cellular senescence and promote anti-tumor immunity, which might represent potential mechanisms for radiosensitization. Several small sample clinical studies have preliminarily indicated that the combination of CDK4/6 inhibitors and radiotherapy exhibited well-tolerated toxicity and promising efficacy. However, most clinical trials in combined therapy remain in the recruitment stage. Further work is required to seek optimal radiotherapy-drug combinations. In this review, we describe the effects and underlying mechanisms of CDK4/6 inhibitors as a radiosensitizer and discuss previous clinical studies to evaluate the prospects and challenges of this combination.

## Background

Radiotherapy is one of the most important local control methods of malignant tumors. Approximately 50% of cancer patients receive radiotherapy during the treatment process [1]. However, the radioresistance of tumor cells limits the ability to reach a curative dose of radiation, which reduces radiotherapy efficacy and is more likely to cause local failures [1, 2]. Substantial efforts have been made to improve the radiosensitivity via various types of radiation modulators. The early-stage radiosensitizers are mostly chemotherapeutic agents, such as cisplatin and 5-fluorouracil, which have been demonstrated to enhance radiosensitivity in head and neck squamous cell carcinoma (HNSCC), nasopharyngeal carcinoma (NPC), and gastrointestinal cancers [3]. These chemotherapeutics exhibit radiosensitization effects by increasing radiation damage and inhibiting DNA repair process [4, 5]. However, due to the non-specific effects of chemotherapeutics, chemoradiation therapy increases the radiation toxicity of normal tissues as well [1]. Given the rapid development of targeted therapies, numerous studies of specific radiosensitizers are underway. CDK4/6 inhibitors are a novel class of selective cell cycle therapeutics that target the cyclin D-CDK4/6 complex and suppress activation of the downstream RB-E2F pathway, thereby blocking cell cycle progression and inhibiting the tumor cell proliferation [6–8]. Multiple preclinical studies have demonstrated the radiosensitization effects of CDK4/6 inhibitors in various cancer types. In this review, we focus on the molecular basis and the underlying mechanisms regarding the efficacy of CDK4/6 inhibitors with radiotherapy to provide a strong rationale for clinical utilization of the combined therapy.

## Overview of CDK4/6 inhibitors

### Role of CDK4/6 in G_1_-S transition

**Fig. 1:**
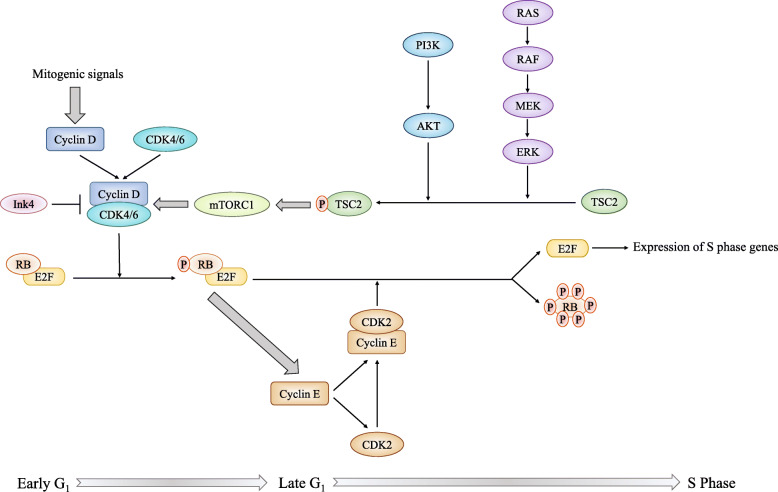
Physiological functions and regulation of the cyclin D-CDK4/6 complex. Cyclin D-CDK4/6 complex facilitates G_1_-S transition. In the early G_1_ phase, mitogenic signals stimulate the accumulation of D-type cyclins and induce the formation of the cyclin D-CDK4/6 complex, which phosphorylates RB. Once hypophosphorylated, RB is prepared for hyperphosphorylation by the increased levels of the cyclin E-CDK2 complex. The transcription factor E2F is released from the RB-E2F complex due to the lack of the inhibitory effects of RB, enabling cells to proceed into S phase. Cyclin D-CDK4/6 activity is also regulated by mTORC1 and Ink4 family proteins. The PI3K and RAS pathway phosphorylate TSC2 to enhance mTORC1 activity, thus increasing cyclin D1 levels. Conversely, Ink4 family proteins inactivate the kinase to block cell cycle progression

**Fig. 2:**
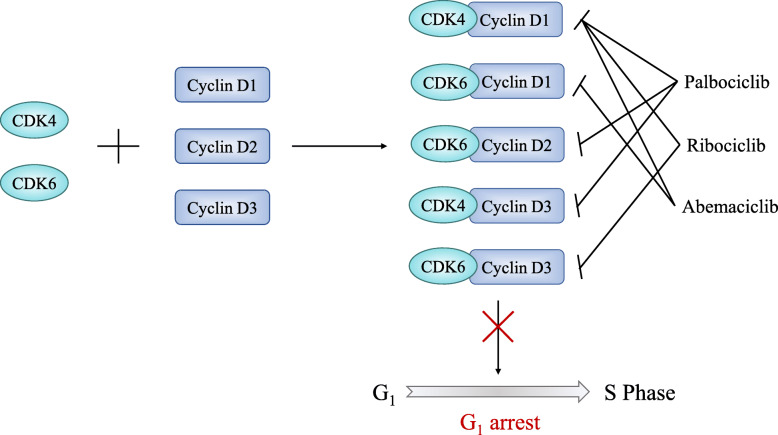
Specific targets of CDK4/6 inhibitors. CDK4/6 inhibitors specifically bind to CDK4/6, thus inducing G_1_ phase arrest. Although these inhibitors perform the same function in cell cycle progression, their targets are slightly different. Palbociclib inhibits cyclin D1-CDK4, cyclin D2-CDK6, and cyclin D3-CDK4. Ribociclib targets cyclin D1-CDK4 and cyclin D3-CDK6. Abemaciclib targets cyclin D1-CDK4/6

CDK4/6 plays a critical role in the G_1_-S checkpoint, which governs genome replication in the cell cycle [9, 10] (Fig. 1). From the classical view, the retinoblastoma (RB) protein, as a negative cell cycle regulator, binds to the transcription factor E2F to repress the transcriptional activity in the early G_1_ phase [9, 11]. Mitogenic stimuli induce increased expression levels of D-type cyclins, which form complexes with CDK4/6 to phosphorylate RB [9]. Hypophosphorylated RB partially relieves the inhibitory control of the E2F transcription factor family; promotes the expression of E2F target genes, such as cyclin E; and facilitates the G_1_ phase progression [9, 11, 12]. In the late G_1_ phase, CDK2 is activated as cyclin E levels increase. The continuous formation of the cyclin E-CDK2 complex hyperphosphorylates and inactivates RB proteins, which results in the release of E2F transcription factors, initiation of transcription, and increased expression of S phase genes [9, 12]. Thereby, the cell cycle proceeds from the late G_1_ to S phase normally. During the G_1_-S transition, CDK4/6 activity is regulated by Cip/Kip (p21^cip1^, p27^kip1^, and p57^kip2^) family and Ink4 family (p16^Ink4a^, p15^Ink4b^, p18^Ink4c^, and p19^Ink4d^) proteins. The Cip/Kip family broadly inhibits the cyclin-CDK complex activity, also known as pan-CDK inhibitors [10, 12]. Conversely, Ink4 family proteins specifically bind to CDK4/6 to inactivate the kinase, thus inducing the inhibition of RB phosphorylation and blockade of cell cycle progression [8, 9, 13]. CDK4/6 inhibitors exert similar tumor-suppressing functions as Ink4 family members [12]. All three CDK4/6 inhibitors bind to the ATP domain of CDK4/6, but their targets are slightly different [14] (Fig. 2).

### Therapeutic applications of CDK4/6 inhibitors

The cyclin D-CDK4/6-RB pathway is frequently dysregulated in many cancers [12]. Moreover, increased levels of D-type cyclins and CDK4 are commonly observed [15–17], which makes inhibitors targeting the cyclin D-CDK4/6-RB pathway ideal candidates for cancer therapeutics. When used as monotherapy, CDK4/6 inhibitors exhibit certain efficacy in solid tumors, such as non-small cell lung cancer (NSCLC) [18, 19] and mature teratoma [20]. Moreover, CDK4/6 inhibitors in combined therapies displayed better prospects. Palbociclib, ribociclib, and abemaciclib have been approved by the Food and Drug Administration for advanced hormone receptor-positive (HR+) breast cancer patients when combined with letrozole or fulvestrant [12, 13]. The combinatorial strategy presented a substantial improvement in progression-free survival with a well-tolerated toxicity profile in multiple clinical trials [21–26]. The combination of CDK4/6 inhibitors and other targeted drugs also shows promising prospects. PI3K-AKT-mTOR and RAS-RAF-MEK-ERK pathway inhibitors both exhibit synergistic tumor suppression effects with CDK4/6 inhibitors in many preclinical models [27–32]. An important mechanism is that activation of oncogenic PI3K and RAS pathways is correlated with the cyclin D-CDK4/6 complex [8, 33]. RAS pathway drives CCND1 mRNA transcription and enhances cyclin D-CDK4/6 activity [34]. Hence, PI3K and RAS pathway inhibitors in combination with CDK4/6 inhibitors produce a double-hit on cyclin D-CDK4/6 activity [9]. Moreover, CDK4/6, PI3K, and RAS pathways intersect via tuberous sclerosis complex 2 (TSC2)(Fig. 1), which is a negative regulator of mammalian target of rapamycin complex 1 (mTORC1) [9, 33]. The PI3K and RAS pathway activate mTOR signaling through phosphorylating TSC2, and CDK4/6 can directly bind to and phosphorylate TSC2 [33, 35]. Therefore, co-inhibition of the PI3K or RAS pathway with the CDK4/6 pathway leads to a synergistic suppression of TSC2 phosphorylation and thus decreases mTORC1 activity [36]. This action might represent another critical mechanism for the synergy of CDK4/6 inhibitors with PI3K or RAS pathway inhibitors.

Although CDK4/6 inhibitors have achieved some clinical success, the lack of predictive biomarkers is a major obstacle preventing CDK4/6 inhibitors from better efficacy. Currently, a majority of research focuses on the potential biomarkers of breast cancer, and the HR-positive status remains the only clinically used biomarker. However, HR expression levels as a biomarker showed no clear survival advantage in the phase III PALOMA-3 trial [13, 25]. Several potential biomarkers, such as CCND1, CDKN2A, and RB1, exhibit an association with the sensitivity of CDK4/6 inhibitors in preclinical studies [12, 13, 37]. The most reliable of these biomarkers is RB1 since amplification of CCND1 and loss of CDKN2A showed no difference in benefit in the phase II PALOMA-1/TRIO-18 study [38]. Loss of RB1 appears to induce resistance of CDK4/6 inhibitors in many in vitro studies, but this finding has yet to be confirmed clinically [12, 37]. Nevertheless, the lack of RB1 expression is rare in ER-positive breast cancer patients, leaving no appropriate biomarkers other than the HR status [39]. However, there are a few ongoing clinical trials specifically targeting the molecular drivers of tumors, such as Lung-MAP (NCT02154490) and SIGNATURE (NCT02187783) trials, in which patients are being allocated based on CCND, CDKN2A, or CDK4 aberrations. More data regarding the verification of various biomarkers will be available after the completion of these ongoing clinical trials.

## Preclinical studies of CDK4/6 inhibitors as radiosensitizers

In addition to intersecting with ER, PI3K, and RAS pathways, the cyclin D-CDK4/6-RB pathway is also involved in DNA damage repair (DDR) [40], which makes CDK4/6 inhibitors perfect candidates for tumor radiosensitization. Numerous in vitro and in vivo data have validated the radiosensitization effects of CDK4/6 inhibitors.

### In vitro experimental data

CDK4/6 inhibitors enhance the radiosensitivity in multiple cell lines. The combination therapy significantly inhibits single-cell colony formation with a sensitizer enhancement ratio (SER) of 1.1–2.3 (Table 1). The radiosensitization effect is quite outstanding in malignant brain cancer cell lines. Two medulloblastoma cell lines, Daoy and ONS-76, were administered different doses of radiation following 48 h of treatment with palbociclib. The enhancement ratio ranged from 1.6 to 2.3 at 10% survival [41]. Moreover, the combination not only decreases colony numbers but also reduces the tumor sphere formation ability [45]. The tumor sphere formation ability is associated with cancer stem cell properties, which includes increased radiation resistance [50, 51]. Huang et al. [45] reported that the combination of 6 Gy radiation and 5 μM palbociclib dramatically reduced tumor sphere formation in Huh7 (hepatocellular carcinoma, HCC) cells compared to ionizing radiation (IR) alone. The combined therapy significantly inhibited radiation-induced cell growth and reduced radiation resistance.

**Table 1:** In vitro experimental data of radiosensitization effects

Authors	CDK4/6 inhibitor	Human cell line	Efficacy with radiotherapy
Whiteway et al. [41]	Palbociclib	Medulloblastoma (Daoy) (ONS-76)	SF_2_^a^ ↓ SER_10_^b^ 1.6, SER_50_^c^ 1.5 SER_10_ 2.3, SER_50_ 2.3
Hashizume et al. [42]	Palbociclib	Intracranial ATRT ^d^ (BT12) (BT16)	DEF ^e^ 1.16–1.60 DEF 1.18–1.70
Whittaker et al. [43]	Palbociclib	Glioblastoma (GBM-L1, HW1, RN1, BAH1)	Colony numbers drop to zero
Naz et al. [44]	Abemaciclib	NSCLC (A549, H460, H820, H1975)	DMF ^f^ 1.30–1.71
Tao et al. [30]	Palbociclib and Trametinib	NSCLC (A549, H460)	Cell survival fraction ↓ Apoptosis ↑
Huang et al. [45]	Palbociclib	HCC (Hep3B, Huh7) CCA ^g^ (HuCCT1)	Tumor sphere numbers ↓ Colony numbers ↓
Xie et al. [46]	Palbociclib	NPC (CNE-1) (CNE-2)	SF_2_ ↓ SER 1.118–1.475 SER 1.10–1.20
Gottgens et al. [47]	Palbociclib	HNSCC (UT-SCC-24A)	SER 1.36–1.60
Tai et al. [48]	Ribociclib	HNSCC (OML1, OML1-R)	Colony numbers ↓ Cell viability ↓
Fernández-Aroca et al. [49]	Palbociclib	Breast cancer (MCF-7) Lung cancer (A549) Colorectal cancer (HCT116)	Cell survival fraction ↓
Li et al. [50]	Ribociclib and CA3	EAC ^h^ (Flo-1 XTR)	Colony numbers ↓

^a^ SF_2_, survival fraction at 2 Gy

^b^ SER_10_, sensitizer enhancement ratio at 10% survival

^c^ SER_50_, sensitizer enhancement ratio at 50% survival

^d^ ATRT, atypical teratoid rhabdoid tumor

^e^ DEF, radiation dose enhancement factor

^f^ DMF, radiation dose modifying factor

^g^ CCA, cholangiocarcinoma

^h^ EAC, esophageal adenocarcinoma

Additionally, although palbociclib, ribociclib, and abemaciclib resemble each other structurally, the three drugs exhibit different radiosensitization effects. Naz et al. [44] demonstrated that palbociclib and ribociclib failed to promote radiation sensitivity in H460 (NSCLC) cells when added either pre- or post-IR treatment. In contrast, abemaciclib exhibited enhancement of radiosensitivity in the majority of NSCLC cell lines when added post-IR, including H460. CDK4/6 inhibitors probably have different radiosensitization effects, and the underlying mechanisms should be assessed in further investigations.

### In vivo experimental data

In vivo preclinical studies have further verified the radiosensitization effects of CDK4/6 inhibitors, including prolonging the median survival time, reducing tumor volume, and delaying tumor regeneration (Table 2). Several in vivo studies demonstrated that palbociclib in combination with IR increased the median survival time by 1.2- to 3.3-fold in mice with brain malignancy xenografts [42, 43, 52]. The combined therapy also dramatically reduced the tumor weight and volume without no obvious systemic toxicity as assessed by mouse body weight [30, 45, 50]. The Ki-67 staining in xenografts was drastically reduced, revealing the reduction of tumor proliferation [30, 44, 50].

**Table 2:** In vivo experimental data of radiosensitization effects

Authors	CDK4/6 inhibitor	Xenograft	Efficacy with radiotherapy
Hashizume et al. [42]	Palbociclib	Intracranial ATRT (BT12) (BT16) Glioblastoma (GBM43)	Median survival time increased by 24 to 26 days (1.4-fold) Median survival time increased by 31.5 to 34.5 days (3.3-fold) Median survival time increased by 10 to 13.5 days (1.6-fold)
Whittaker et al. [43]	Palbociclib	Glioblastoma (RN1)	Median survival time increased by 8 days
Naz et al. [44]	Abemaciclib	NSCLC (H460)	Tumor regrowth delay 8 and 9 days, inhibit IR-induced tumor vasculogenesis
Tao et al. [30]	Palbociclib and Trametinib	NSCLC (A549)	Tumor volume ↓ Proliferation ↓ Apoptosis ↑
Huang et al. [45]	Palbociclib	HCC (Huh7)	Tumor volume ↓ Tumor weight ↓
Li et al. [50]	Ribociclib and CA3	EAC (Flo-1 XTR)	Tumor volume ↓ Tumor weight ↓
Barton et al. [52]	Palbociclib	Ink4a-ARF- deficient BSG ^a^ mouse model	Median survival time increased by 10 days (19%)

^a^ BSG, brainstem glioma

## Mechanisms of CDK4/6 inhibitors as radiosensitizers

### Inhibition of DNA damage repair

The impairment of DDR is an essential determinant of CDK4/6 inhibitor-associated radiosensitization in human cell lines. IR produces cytotoxic effects on DNA, which mainly results in DNA single-strand breaks (SSBs) and double-strand breaks (DSBs) [53]. Unrepaired and inaccurate-repaired DNA DSBs are the main cause of radiation-induced cell death [54]. Multiple preclinical studies have demonstrated that IR increases γ-H2AX and 53BP1 levels in a dose dependent manner and increases DNA DSBs [30, 42, 44–47]. γ-H2AX is one of the earliest events of DDR with the induction of DSB [55]. The abundance of γ-H2AX foci reaches a peak at 30 min after IR and returns to baseline levels approximately 24 h post-IR [37]. In addition, 53BP1 plays a similar role to γ-H2AX in most cases [45], which is also a crucial hallmark of IR-induced DSBs [56]. Naz et al. [44] investigated γ-H2AX foci in H460 and H1299 (NSCLC) cells following 7.5 Gy irradiation. The γ-H2AX level rapidly increased in H460 (7.9-fold) and H1299 (6.7-fold) cells 0.5 h after IR compared to untreated control. Additional studies also demonstrated that various post-irradiated cell lines showed substantial increases in γ-H2AX and (or) 53BP1 foci, which eventually led to increased DSBs [37, 42, 45–47, 49].

γ-H2AX and 53BP1 levels decrease over time, reflecting the dynamic process of DDR. The combination of IR and CDK4/6 inhibitors causes marked retention of γ-H2AX and (or) 53BP1 levels, thus delaying DSB repair to enhance radiotherapy efficacy [42, 44–47]. With 7.5 Gy and 10 μM abemaciclib, γ-H2AX levels were increased 3.92-fold at 24 h in H460 cells. However, the levels were only 0.97-fold in H1299 cells (radiation-resistant), which was basically equivalent to the level before irradiation [44]. Huang et al. [45] reported similar findings in HuCCT1 (CCA), Huh7, and Hep3B (HCC) cells. These post-IR cells treated with 5 μM palbociclib sustained higher levels of γ-H2AX and 53BP1 at 24 h compared with DMSO-treated cells. Several other studies also supported this view that CDK4/6 inhibitors exhibited cellular radiosensitivity by increasing unrepaired DSBs and inducing the delayed repair kinetics of DSBs [42, 46, 47].

The combination of CDK4/6 inhibitors and IR not only increases DSBs and delays the DSB repair but also causes homologous recombination (HR) deficiency by decreasing the expression of Rad51 and ataxia telangiectasia mutated (ATM) kinase [57]. HR and non-homologous end joining (NHEJ) are the two principal pathways in DSB repair, and the latter plays a dominant role in IR-induced DDR [55, 58]. The DNA recombinase Rad51 is a pivotal component of the HR pathway, and the fraction of Rad51 foci increases during the HR process [47]. ATM kinase, the upstream regulator of Rad51, is activated to enhance HR in response to DSBs [55, 57]. In UT-SCC-24A (HNSCC) cells, palbociclib combined with IR reduced the expression of Rad51 approximately 3.5-fold [47]. Palbociclib also impaired ATM kinase activation by reducing phosphorylation of ATM kinase and its downstream targets with 10 Gy irradiation in both HCC and CCA cells [45]. However, impairment of the HR pathway appeared to be dependent on a functional p53 or RB status. Abemaciclib and IR decreased the formation of Rad51 foci markedly in H460 (p53-proficient) cells. Nevertheless, no significant changes were observed in H1299 (p53-deficient) cells [44]. A possible explanation was that p53 is a key effector in IR-induced cellular response and the lack of p53 was associated with increased radioresistance [59]. This hypothesis was confirmed by Fernández-Aroca et al. [49], who reported that p53 was a critical determinant of palbociclib-associated radiosensitivity. Dean et al. [37] shared similar findings in breast cancer given that the response of Rad51 to palbociclib with IR presented in an RB-dependent manner. Palbociclib pretreatment led to complete inhibition of Rad51 foci accumulation in MDA-MB-231 and Hs578t (RB-proficient) cells but not in MDA-MB-468 (RB-deficient) cells. Furthermore, 500 nM palbociclib caused an approximately 60% decrease in HR-mediated DSB repair and 2.5-fold increase in relative NHEJ activity, indicating that CDK4/6 inhibition augmented NHEJ efficiency [37]. However, current data are not available to demonstrate that CDK4/6 inhibitors with IR enhance NHEJ activity while weakening the HR efficiency. More studies are currently underway to elucidate this relationship.

### Enhancement of apoptosis

As mentioned above, radiotherapy induces massive DSBs. As the intracellular stimulus, IR-induced DNA damage mediates most radiosensitivity-associated pro-apoptotic effects [60]. Several preclinical studies investigated apoptotic changes in combined therapy. Huang et al. [45] showed that 8 Gy irradiation plus 20 μM palbociclib resulted in remarkably increased DNA fragmentations (a hallmark of apoptosis) in HCC and CCA cells compared with monotherapy. Another study also reached similar conclusions in NPC cells, demonstrating that palbociclib treatment after irradiation prominently elevated the proportion of apoptotic cells in comparison with IR alone (CNE-1, 19.6% versus 10.685%; CNE-2, 21.655% versus 12.635%). Mechanistically, they demonstrated that the combination augmented the mitochondrial reactive oxygen species (ROS) level, which is regarded as an apoptosis mediator in radiotherapy or chemotherapy, thus enhancing apoptosis [46]. Interestingly, Hagen et al. [61] reported that palbociclib induced anti-apoptosis effects in irradiated MCF10A (normal human mammary epithelial cell) and MDA-MB-231 (triple-negative breast cancer, TNBC) cells by reducing cleaved PARP levels. The reason why the investigators reached the opposite conclusion remains unclear, but the radiotherapy-drug combination strategy and the p53 status of different cell lines could be an explanation.

### Blockade of cell cycle progression

Another critical determinant of radiosensitivity is cell cycle arrest since CDK4/6 inhibitors induce significant G_1_-S arrest [11]. Meanwhile, G_2_-M phase cells are the most sensitive to radiation [4]. Tai et al. [48] found that 4 Gy irradiation in combination with ribociclib caused evident G_1_-S arrest in HNSCC cells as the ratio of OML1 cells in the G_1_ phase increased from 48.6 to 69.4% after treatment and similar effects were observed in radioresistant OML1-R cells. Xie et al. [46] demonstrated that concurrent palbociclib with IR and radiotherapy followed by palbociclib in NPC cells conspicuously increased the G_2_-M cell proportion and decreased radioresistant G_1_ cells, suggesting that the combination therapy also caused G_2_-M arrest. Furthermore, the combined regimens not only blocked G_1_-S and G_2_-M checkpoint but also suppressed mitosis. Gottgens et al. [47] investigated the changes in phosphorylated histone 3 (p-H3) at Ser10 in UT-SCC-24A (HNSCC) cells in response to IR and palbociclib. Phospho-H3 (Ser10) is associated with chromosome condensation, which is a major event during mitosis [62]. After IR alone, p-H3 (Ser10) levels dropped rapidly and returned to normal at 24 h. In contrast, p-H3 (Ser10) depleted quickly without rebound after combing palbociclib and IR, indicating a decreased number of mitotic cells and inhibition of mitosis. Intriguingly, Hagen et al. [61] reported that knockdown of CDK4 did not affect cell cycle as no marked changes in cell cycle distribution were observed after IR in shCDK4 cells. A possible explanation is that silencing CDK4 and CDK4/6 inhibitors display different outcomes in terms of cell cycle progression. In general, CDK4/6 inhibitors in combination with IR provide a strong blockade of cell cycle progression, which is a critical mechanism of radiosensitivity.

### Other potential mechanisms

In addition to the classic radiosensitization mechanisms mentioned above, CDK4/6 inhibitors also induce other biological phenotypes, providing mechanistic foundations for the combination of CDK4/6 inhibitors and radiotherapy.

First, long-term exposure to CDK4/6 inhibitors induces the cellular senescence phenotype in many cancer cell lines, such as breast cancer [63], neuroblastoma [64], and melanoma [65]. It is not surprising as CDK4/6 inhibitors play a similar role to p16^Ink4a^, and the p16^Ink4a^-RB pathway is one of the most important mechanisms of cellular senescence [66, 67]. Persistent CDK4/6 inhibition suppresses RB phosphorylation and downstream transcription activities to induce an irreversible arrest of cell proliferation, which is also known as cellular senescence [68, 69]. After 8 days exposure of palbociclib, the fraction of senescence-associated β-galactosidase (SA-β-gal)-positive cells was significantly increased in 1205Lu (melanoma) cells [65]. Ribociclib also yielded similar results in neuroblastoma cell lines [64]. On the other hand, radiotherapy triggers premature senescence in solid tumor cell lines [70]. This finding is attributed to the fact that IR causes massive lesions in DNA, therefore activates the ATM-Chk2-p53-p21 axis (senescence-associated DDR pathway), leading to persistent cell cycle arrest and cellular senescence [68]. In addition, 8 or 10 Gy irradiation accelerated cellular senescence in TP53 wild-type tumor cell lines based on increased SA-β-gal positivity [70, 71]. Accordingly, radiotherapy and CDK4/6 inhibitors may synergistically induce tumor cell senescence and further inhibit tumor progression.

Senescent cells secrete inflammatory cytokines, chemokines, and growth factors, which collectively comprise the the senescence-associated secretory phenotype (SASP) [67, 68]. After treatment with palbociclib for 8 days, SASP components, such as interleukin-6 (IL-6), interleukin-8 (IL-8), and chemokine (C-X-C motif) ligand 1 (CXCL1), secreted by 1205Lu cells substantially increased [65]. Furthermore, radiation triggers the release of cytoplasmic DNA and activates the cyclic GMP-AMP synthase (cGAS)-stimulator of interferon genes (STING) pathway, which plays a pivotal role in SASP production [72, 73]. Thus, the combination of CDK4/6 inhibitors and radiation could theoretically induce SASP, which affects the immune response [72]. On one hand, SASP recruits immune cells to stimulate the adaptive immune response and eliminate senescent tumor cells. On the other hand, SASP also attracts immunosuppressive cells and creates a protumorigenic environment [72]. Although SASP is usually regarded as a “double-edged sword”, it still provides a novel perspective for radiosensitization mechanisms. Thus, the combination of CDK4/6 inhibitors and radiotherapy may exert radiosensitization effects through immunomodulation.

Indeed, several investigators have demonstrated that CDK4/6 inhibitors exhibited direct immunostimulatory effects in both tumor and immune cells [74]. In tumor cells, CDK4/6 inhibitors suppressed the RB-E2F-DNMT1 axis, which activated endogenous retroviral elements and increased double-stranded RNA levels. This action subsequently induced a type III interferon response and upregulated tumor antigen presentation [75]. On the other hand, radiotherapy enhances MHC class I expression by activating the mTOR pathway [76]. Moreover, radiation elicits activation of dendritic cells (DCs) and enhances cross-presentation of tumor antigens [73, 76]. Gupta et al. [77] reported that the expression of CD70 and CD86 (co-stimulatory molecules) on DCs was significantly increased after 10 Gy irradiation. In immune cells, CDK4/6 inhibitors promoted T cell activation via enhancing nuclear factor of activated T cells (NFAT) transcriptional activity and interleukin-2 (IL-2) production [78, 79]. On the other hand, radiation-induced T cell activation has been demonstrated in several preclinical studies, which is mediated by the induction of viral mimicry and activation of the cGAS-STING pathway [80–82]. Although IR recruits regulatory T cells (Treg) and other immunosuppressive cells to the tumor microenvironment, CDK4/6 inhibitors markedly reduce the proliferation of Tregs [75]. Collectively, CDK4/6 inhibitors and radiotherapy may synergistically exert an anti-tumor immune response by enhancing antigen presentation capacity and T cell activation. These potential mechanisms offer new perspectives for future exploration. The combination of CDK4/6 inhibitors and radiation may not only improve local tumor control but also enhance systemic disease control, providing the possibility for the triplet combination of CDK4/6 inhibitors, radiotherapy and immunotherapy.

## Clinical studies of CDK4/6 inhibitors in combination with radiotherapy

Multiple preclinical data suggest a potential synergistic effect when CDK4/6 inhibitors and radiotherapy are administered concurrently. However, safety and efficacy still require further investigations. Currently, there are six ongoing clinical trials (Table 3) as well as several small sample clinical studies (Table 4), which mostly target HR+/HER2- metastatic breast cancer (MBC) patients. The preliminary results [83–86] propose that no significantly increased toxicity is observed, indicating that the combined therapy is promising as a novel strategy. Ippolito et al. [84] reported that hematological toxicity neutropenia is most common among all adverse events. In total, 60% of patients experienced grade 3 or greater neutropenia before combination treatment, suggesting that we should carefully evaluate previous toxicity to prevent reoccurrence. Figura et al. [85] retrospectively analyzed 42 lesions in 15 brain MBC patients. Two lesions (5%) developed radiation necrosis, and both of them underwent four previous RT courses before the occurrence of radionecrosis, indicating the significance of cautious assessment of the treatment plan margins. Chowdhary et al. [86] reported that concomitant treatment with palbociclib and RT resulted in grade 1 or 2 toxicity and notably relieved pain. Also, no local failure was noted in evaluable follow-up patients.

**Table 3:** Clinical trials with CDK4/6 inhibitors in combination with IR

Cancer Type	Phase	Arm	n	Status	NCT
HGG ^a^, DIPG^b^, bithalamic HGG	I/II	Ribociclib + IR	24	Active, not recruiting	NCT02607124
Glioma (HGG, DIPG et al.)	I	Ribociclib + Everolimus + IR	24	Recruiting	NCT03355794
Locally advanced HNSCC	I/II	Palbociclib + Cetuximab + IMRT ^c^	33	Recruiting	NCT03024489
HPV-unrelated HNSCC	II	Palbociclib + Cetuximab or Cisplatin + IMRT	29	Recruiting	NCT03389477
Bone metastatic breast cancer (HR+/HER2-)	II	Palbociclib + Hormone therapy + IR	42	Recruiting	NCT03691493
Metastatic breast cancer (HR+/HER2-)	II	Palbociclib + Letrozole ± SBRT ^d^	204	Not yet recruiting	NCT04220476

^a^ HGG, high-grade glioma

^b^ DIPG, diffuse intrinsic pontine glioma

^c^ IMRT, intensity-modulated radiation therapy

^d^ SBRT, stereotactic body radiation therapy

**Table 4:** Case reports and clinical studies with CDK4/6 inhibitors in combination with IR

Authors	Patients	Arm	Toxicity	Efficacy
Hans et al. [83]	Metastatic breast cancer (HR+/HER2-) *n* = 5	Palbociclib + Fulvestrant + palliative IR	Digestive toxicity: mucositis (grade 1 = 20%, grade 2 = 20%) Hematological toxicity: grade 3 neutropenia = 40%, grade 3 anemia = 20%, grade 3 thrombopenia = 40% No skin toxicity	Symptom control and pain relief (100%)
Ippolito et al. [84]	Metastatic breast cancer (HR+) *n* = 16	Palbociclib or Ribociclib + palliative IR	Hematological toxicity: neutropenia (grade 2 = 12.5%, grade 3 = 25%, grade 4 = 6.3%)	Absent
Figura et al. [85]	Brain metastatic breast cancer (HR+) *n* = 15 lesions = 42	Palbociclib or Abemaciclib + SBRT	Radionecrosis (2 lesions, 5%) No neurologic toxicity or scalp toxicity	Median OS 36.7 months Six-month local control (88%) Six-month distant brain control (61%)
Chowd-hary et al. [86]	Metastatic breast cancer (HR+/HER2-) *n* = 16	Palbociclib + Fulvestrant or Letrozole + palliative IR	Hematological toxicity: grade 1 = 87.5%, grade 2 = 12.5% No grade 2 or higher cutaneous, neurologic, or gastrointestinal toxicity	Local control and pain relief (100%)

Given these results, the combined treatment seems well-tolerated, whereas severe adverse effects may be a concern. A breast cancer patient with supraclavicular lymph node metastasis was reported to develop grade 3 radiation esophagitis and dermatitis after receiving palbociclib with 40 Gy in 20 fractions to the left neck [87]. Another case was a breast cancer patient with bone metastases at the left iliac bone and first sacral vertebrae. This patient experienced radiation-induced grade 3 enterocolitis after administration of palbociclib and 30 Gy in 10 fractions to pelvic bones, which might be related to the over-sensitization after palbociclib administration [88]. Hence, combination therapy should be used cautiously until more data are available. In addition, the patient’s condition should be thoroughly evaluated and treated individually before the utilization of combination regimens.

## Conclusions

CDK4/6 inhibitors have greatly changed the treatment landscape of HR-positive breast cancer patients, stimulating further explorations of combination therapy. Significant preclinical data have demonstrated the radiosensitization effects of CDK4/6 inhibitors via inhibiting DDR, enhancing apoptosis, and blocking cell cycle progression. CDK4/6 inhibitors also induce cellular senescence and promote anti-tumor immunity, which might be potential mechanisms for clinical radiosensitization. Although several clinical studies have presented well-tolerated toxicity and promising efficacy for combination therapy, safety remains a major concern. Radiotherapy is currently used as palliative treatment in clinical trials, and most patients have already received irradiation in previous treatment processes. Therefore, re-irradiation is likely to cause cumulative toxicity and severe adverse events. In addition to palliative treatment, radiotherapy can also be used as a postoperative adjuvant treatment in combination with CDK4/6 inhibitors. Several studies demonstrated that CDK4/6 inhibitors were not only efficacious for advanced or metastatic breast cancer patients but also showed promise for early breast cancer patients [89, 90]. Thus, combination therapy still exhibits promising application prospects despite the risks of severe adverse events. In summary, our data provide a strong rationale for the clinical application of CDK4/6 inhibitors as radiation modifiers. However, more work remains to be done to achieve optimal clinical impacts.

## Data Availability

Not applicable.

## Abbreviations

ATM: Ataxia telangiectasia mutated
ATRT: Atypical teratoid rhabdoid tumor
BSG: Brainstem Glioma
CCA: Cholangiocarcinoma
CDK4/6: Cyclin-dependent kinases 4 and 6
cGAS: Cyclic GMP-AMP synthase
CXCL1: Chemokine (C-X-C motif) ligand 1
DC: Dendritic cell
DDR: DNA damage repair
DEF: Radiation dose enhancement factor
DIPG: Diffuse intrinsic pontine glioma
DMF: Radiation dose modifying factor
DSB: Double-strand break
EAC: Esophageal adenocarcinoma
HCC: Hepatocellular carcinoma
HGG: High grade glioma
HNSCC: Head and neck squamous cell carcinoma
HR: Homologous recombination
IL-2: Interleukin-2
IL-6: Interleukin-6
IL-8: Interleukin-8
IMRT: Intensity modulated radiation therapy
IR: Ionizing radiation
MBC: Metastatic breast cancer
mTORC1: Mammalian target of rapamycin complex 1
NFAT: Nuclear factor of activated T cells
NHEJ: Non-homologous end joining
NPC: Nasopharyngeal carcinoma
NSCLC: Non-small cell lung cancer
p-H3: Phosphorylated histone 3
RB: Retinoblastoma
ROS: Reactive oxygen species
SA-β-gal: Senescence-associated β-galactosidase
SASP: Senescence-associated secretory phenotype
SBRT: Stereotactic body radiation therapy
SER: Sensitizer enhancement ratio
SER_10_: Sensitizer enhancement ratio at 10% survival
SER_50_: Sensitizer enhancement ratio at 50% survival
SF_2_: Survival fraction at 2 Gy
SSB: Single-strand break
STING: Stimulator of interferon genes
TNBC: Triple-negative breast cancer
Treg: Regulatory T cells
TSC2: Tuberous sclerosis complex 2
